# Immediate extubation after single-stage laryngotracheal reconstruction for subglottic stenosis in children

**DOI:** 10.1007/s00405-023-07858-5

**Published:** 2023-02-02

**Authors:** XinYe Tang, Yang Yang, ZhiHai Zhang, Rong Sun

**Affiliations:** 1 Department of Otolaryngology, Children’s Hospital of Chongqing Medical University, Chongqing, China; 2 Ministry of Education Key Laboratory of Child Development and Disorders, Chongqing, China; 3 National Clinical Research Center for Child Health and Disorders (Chongqing), Chongqing, China; 4 China International Science and Technology Cooperation Base of Child Development and Critical Disorders, Chongqing, China; 5 Department of Physical Examination, The First Affiliated Hospital of Chongqing Medical University, No. 1, Youyi Road, Yuzhong District, Chongqing, 400016 China

**Keywords:** Laryngotracheal reconstruction, Subglottic stenosis, Costal cartilage, Graft, Stent

## Abstract

**Purpose:**

To evaluate the feasibility and clarify the appropriate indications for extubation immediately after single-stage laryngotracheal reconstruction (SS-LTR) in pediatric subglottic stenosis (SGS).

**Methods:**

A retrospective study was performed from July 2017 to July 2022. All patients underwent SS-LTR with anterior costal cartilage graft. Information such as demographics, comorbidities, history of intubation or tracheostomy, Classification and grading of airway stenosis, the operation-specific decannulation rate and overall decannulation rate were analyzed.

**Results:**

Twenty-two patients with simple SGS were identified. The median age at SS-LTR was 19 months (IQR = 18.5 months). Fourteen patients (63.6%) were intubated prior to the presentation of symptoms. Fourteen patients (63.6%) required preoperative tracheostomy to maintain a secure airway. Eight patients (36.4%) had congenital SGS, 10 patients (45.5%) had acquired SGS, and 4 patients (18.2%) had mixed SGS. Three patients had Grade II stenosis. Nineteen patients had Grade III stenosis. Comorbidities were found in 10 patients (45.5%). Major comorbidities were pneumonia. Congenital airway anomalies were found in 6 patients (27.3%). After anesthesia, all 22 patients were successfully extubated and returned to the general ward. Twenty patients had a satisfactory airway after SS-LTR. Two patients required reintubation or tracheostomy after operation. Operation-specific decannulation rate was 90.9%. The overall decannulation rate is 100%.

**Conclusion:**

SS-LTR with anterior costal cartilage graft is an effective method to treat simple SGS ranging from Grades I to III in children. Extubation immediately after surgery is safe and feasible.

## Introduction

Laryngotracheal reconstruction (LTR) is an effective method used for the treatment of subglottic stenosis (SGS) in children [1]. LTR was first reported by Fearon and Cotton in 1972 [2]. The procedure often involves a postoperative tracheostomy and the placement of an intraluminal stent for several months, which is known as double-stage LTR (DS-LTR). However, tracheostomy and long-term stenting may cause certain potential complications, including granulation tissue, infection and dislodgment of the stent. Occasionally, several surgeries may be required to extubate and remove the granulation tissue in the airway [3]. To solve the aforementioned issues as effectively as possible, a single-stage LTR (SS-LTR) technique was developed [4, 5]. Single-stage surgery allows for tracheostomy decannulation following LTR. However, an endotracheal tube must be placed in the airway as a stent for 0–14 days. Accompanying disadvantages of the single-stage technique include a longer treatment period in the pediatric intensive care unit, a higher level of postoperative sedation and a higher risk of emergent reintubation than the double-stage technique [6]. The endotracheal tube can be removed immediately following SS-LTR and the patient can then be returned to the general ward; this may prove beneficial and may resolve the aforementioned disadvantages. However, related studies on this subject are limited.

The present study analyzed the clinical characteristics and surgical outcomes of patients undergoing SS-LTR, with the aim of evaluating the feasibility and elucidating the indications for extubation immediately following SS-LTR in pediatric SGS.

## Materials and methods

The present study reviewed the medical records of patients who underwent SS-LTR at our institution during the period between July 2017 and July 2022. All pediatric patients had SGS. The site of stenosis was confined only to the subglottis, which does not involve the glottic structures or trachea. The preoperative severity of airway stenosis was graded according to the Myer–Cotton airway grading system (Grade I, 0–50% obstruction; Grade II, 51–70% obstruction; Grade III, 71–99% obstruction; Grade IV, no detectable lumen) [7]. The vertical length of SGS can be measured during the surgery. Information such as age, sex, gestational age, history of intubation and tracheostomy, comorbidities, findings on airway endoscopy, complications, the operation-specific decannulation rate and overall decannulation rate was collected.

**Fig. 1:**
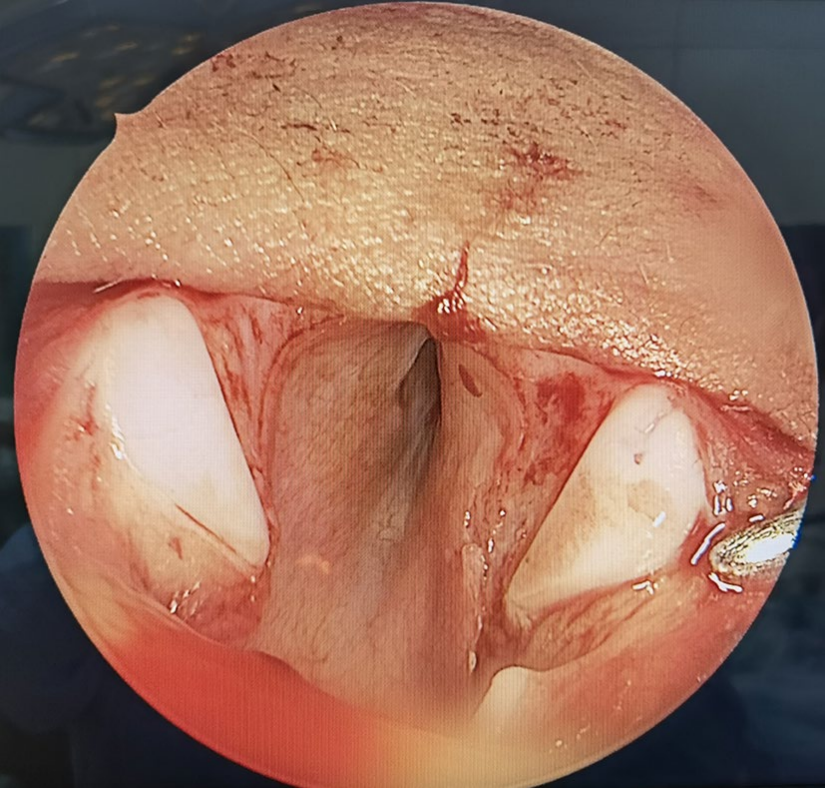
Significantly thickened cricoid arch

**Fig. 2:**
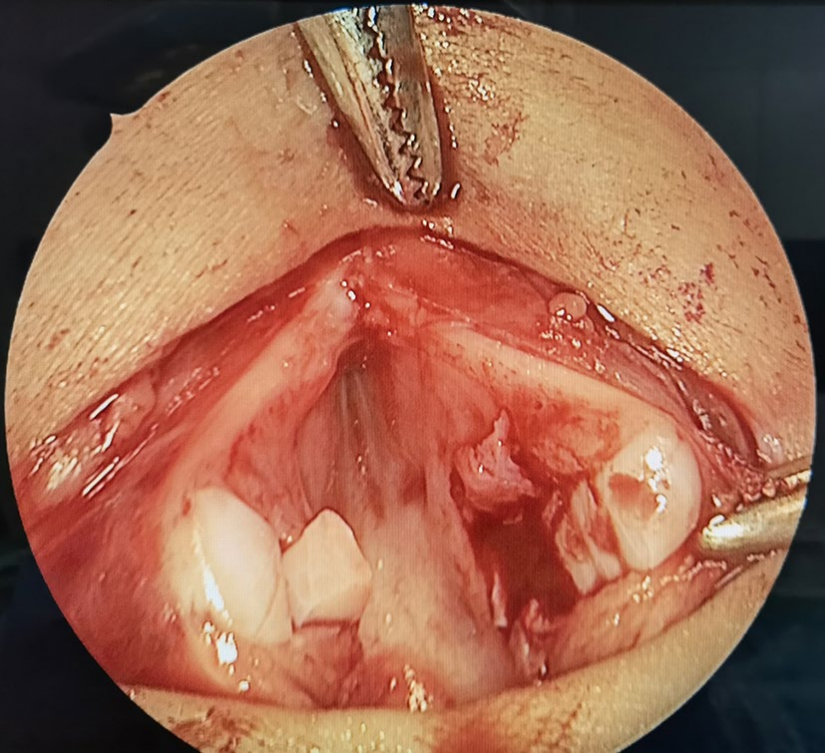
Thickened cricoid arch were partially resected

**Fig. 3:**
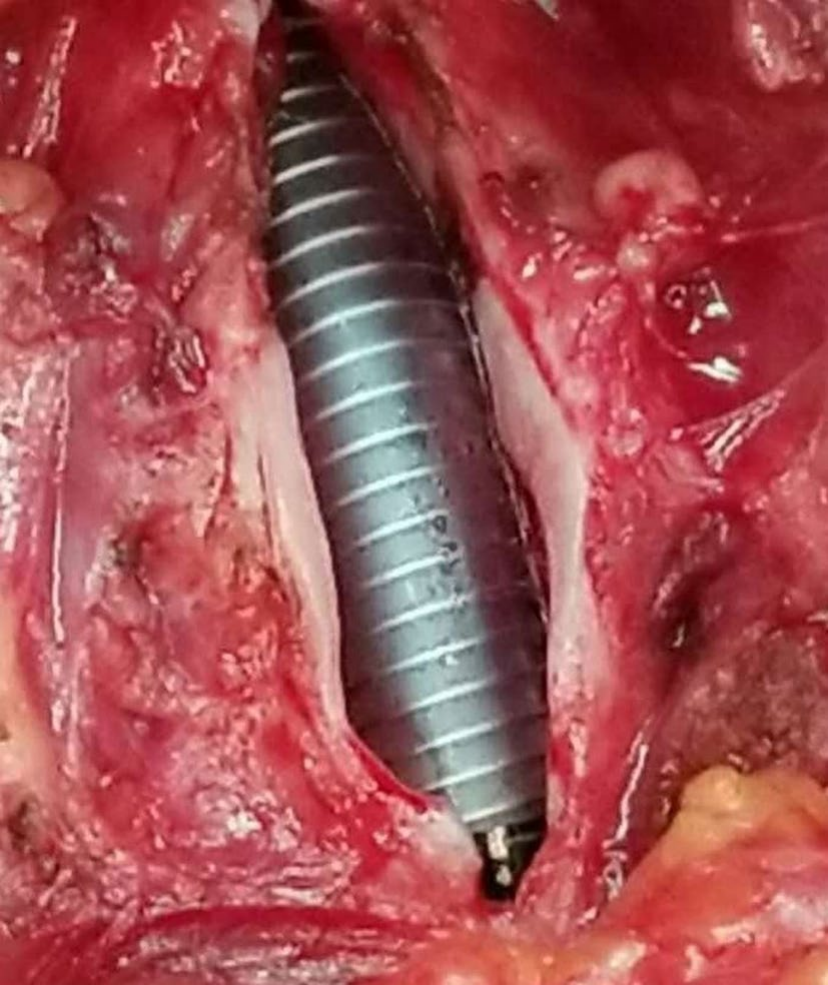
An age-appropriate-sized endotracheal tube was placed for expanding the airway to its normal size

**Fig. 4:**
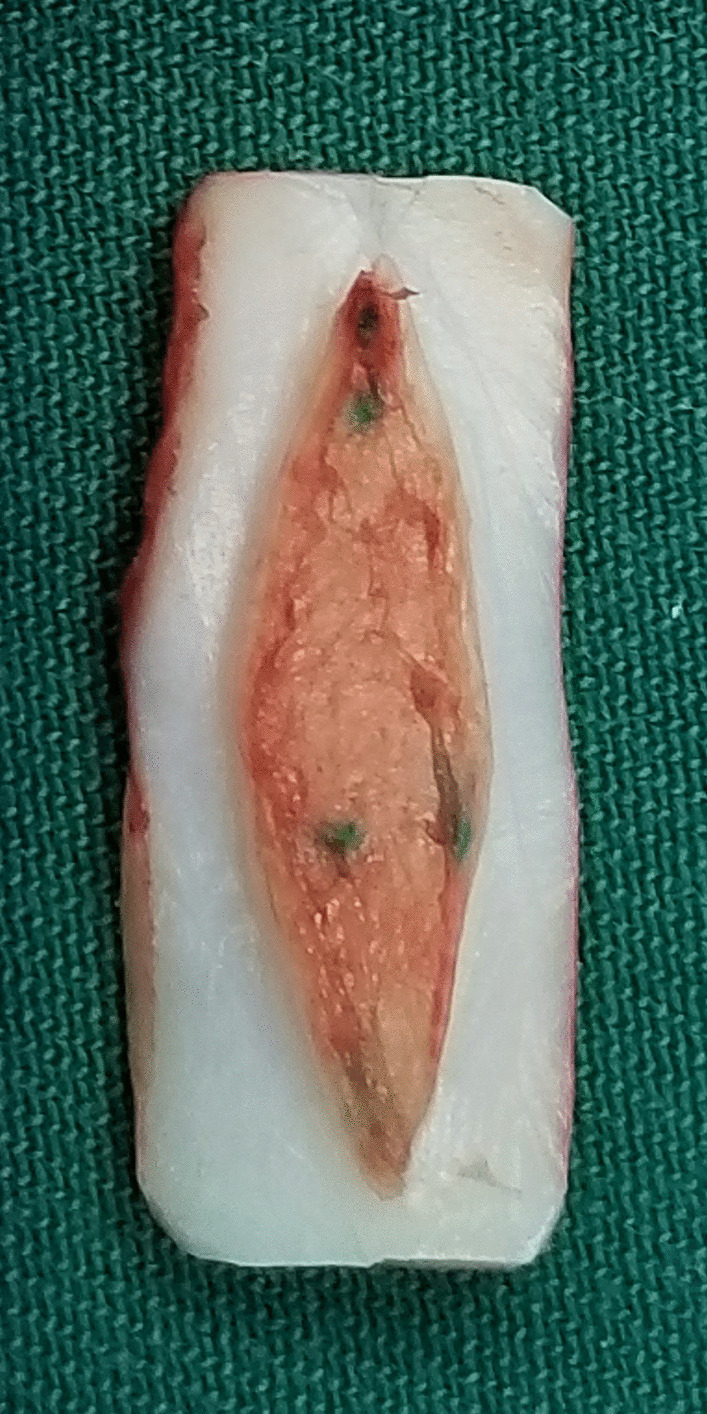
The harvested costal cartilage was carved to a boat-shaped graft with lateral flanges

SS-LTR with anterior costal cartilage graft was used to treat all the patients. The main surgical steps were as follows: after performing a vertical midline anterior incision of the airway, the submucosal scar tissue and/or the excess cartilage of the cricoid ring were partially or totally resected (Figs. 1, 2). An age-appropriate-sized endotracheal tube was then placed for expanding the airway to its normal size (Fig. 3). The oval defect in the anterior subglottis and trachea was the exact size of the graft required. The harvested costal cartilage was carved to a boat-shaped graft with lateral flanges (Fig. 4). The costal cartilage graft was secured in the oval defect in the anterior subglottis and trachea with 4–0 absorbable sutures. If present, the tracheostoma was closed.

The endotracheal tube was removed immediately following anesthetic resuscitation and the pediatric patients were returned directly to the general ward after surgery. Antibiotics were commenced prior to surgery according to the sputum drug sensitivity test and were continued for at least 7 days after the surgery. Systemic glucocorticoids were administered for 3 days. Pulmicort was administered as an aerosol 3 times a day during the hospitalization period. The patients were fed using a gastric tube as early as possible after surgery and switched to oral feeding 7 days later. Proton pump inhibitors were routinely administered for at least 6 weeks. All the patients underwent rigid bronchoscopy on the postoperative day 7. If the results of airway reconstruction appeared satisfactory, the patient was discharged. Follow-up is required for at least 3 months.

Numerical data were presented by median (interquartile range, IQR) and categorical data were presented by percentage (95% confidence interval, CI). All data were analyzed using IBM SPSS (version 25.0, SPSS Institute, Chicago, USA).

The institutional review board of the Children’s Hospital of Chongqing Medical University gave expedited approval of this protocol (File No. 2021251). The study was complied with the Helsinki Declaration of 1975 (revised in 2008).

## Results

A total of 22 patients (15 male and 7 female) who had simple SGS were included in the present study. Seven patients (31.8%, 95% CI 10.7–53.0%) were born preterm (< 37 weeks of gestation). The median age at SS-LTR was 19 months (IQR 18.5 months). Fourteen patients (63.6%, 95% CI 41.8–85.5%) were intubated prior to the presentation of symptoms. The median duration of intubation was 7.0 days (QR 15.5 days). Fourteen patients (63.6%, 95% CI 41.8–85.5%) required preoperative tracheostomy to maintain a secure airway. Eight patients (36.4%, 95% CI 14.5–58.2%) had congenital SGS, 10 patients (45.5%, 95% CI 22.9–68.1%) had acquired SGS, and 4 patients (18.2%, 95% CI 0.7–35.7%) had mixed SGS (which results from intubation on congenital SGS). According to the Myer–Cotton classification system, 3 patients (13.6%, 95% CI 4.7–29.2%) had Grade II stenosis, 19 patients (86.4%, 95% CI 70.8–95.3%) had Grade III stenosis, and none of the patients had Grade IV stenosis. The median vertical length of SGS was 6.0 mm (IQR 2.25 mm). Four patients (18.2%, 95% CI 0.7–35.7%) were treated with balloon dilation for 2–3 times prior to SS-LTR. The stenosis recured to its original Grade after balloon dilation. The other 18 patients (81.8%, 95% CI 64.3–99.3%) had no a history of previous airway surgery for stenosis prior to SS-LTR. Comorbidities were found in 10 patients (45.5%, 95% CI 22.9–68.1%) (Table 1). Major comorbidities were pneumonia. Four patients (18.2%, 95% CI 0.7–35.7%) had multiple comorbidities. These comorbidities were effectively controlled prior to surgery. Congenital airway anomalies were found in six patients (27.3%, 95% CI 7.1–47.5%): two patients (9.1%, 95% CI 0.2–22.1%) had tracheomalacia, two patients (9.1%, 95% CI 0.2–22.1%) had mild laryngomalacia, two patients (9.1%, 95% CI 0.2–22.1%) had pharyngomalacia, one patient (4.5%, 95% CI 0–13.4%) had mild laryngeal web, 1 patient (4.5%, 95% CI 0–13.4%) had mild right main bronchial stenosis.

**Table 1:** Characteristics of patients with subglottic stenosis

Patient no./Sex/Age, mo	Congenital anomalies	Comorbidities	Classification	Vertical length, mm
1/M/48	Right MBS Right thumb polydactyly Gallbladder absence	None	Mixed	5
2/F/26	None	Pneumonia	Acquired	6
3/M/84	Laryngeal web Congenital penile torsion	None	Congenital	8
4/F/25	None	None	Congenital	7
5/F/11	Congenital jejunal atresia Atrial septal defect	Pneumonia Severe malnutrition GER	Congenital	6
6/M/20	None	AIDS	Acquired	7
7/F/6	None	None	Acquired	6
8/M/8	None	None	Mixed	7
9/M/40	None	None	Acquired	7
10/F/21	None	None	Acquired	7
11/M/18	Laryngomalacia	Pneumonia	Congenital	5
12/M/6	Laryngomalacia PDA	Pneumonia GER	Acquired	6
13/F/26	None	Epilepsy	Acquired	8
14/M/24	None	None	Acquired	8
15/M/1	None	Pneumonia GER	Congenital	5
16/M/9	None	Pneumonia	Acquired	6
17/M/12	None	None	Mixed	6
18/M/6	Tracheomalacia Pharyngomalacia	None	Congenital	8
19/M/26	Tracheomalacia Pharyngomalacia	Pneumonia GER	Acquired	9
20/F/3	None	None	Congenital	4
21/M/8	None	Pneumonia	Congenital	4
22/M/59	None	None	Mixed	5

*AIDS* acquired immunodeficiency syndrome, *GER* gastroesophageal reflux, *MBS* Main Bronchial Stenosis, *PDA* patent ductus arteriosus

Following anesthesia, all 22 patients were successfully extubated and were returned to the general ward. Twenty patients (90.9%, 95% CI 77.9–99.8%) had a satisfactory reconstructed airway following SS-LTR with anterior costal cartilage graft. Of those 20 patients, 7 patients (35.0%, 95% CI 12.1–57.9%) had no stenosis, and 13 patients (65.0%, 95% CI 42.1–87.9%) had asymptomatic Grade I stenosis (Table 2). The surgery-specific decannulation rate was 90.9% (95% CI 77.9–99.8%). Two patients (9.1%, 95% CI 0.2–22.1%) required reintubation or tracheostomy after SS-LTR. Patient 2 was reintubated due to severe pneumonia on postoperative day 7. The endotracheal tube was successfully removed after 2 weeks of treatment in the pediatric intensive care unit. The patient had no discomfort at rest; however, normal physical activity caused mild dyspnea following extubation. Scar tissue hyperplasia was founded at the subglottis on rigid bronchoscopy. The degree of stenosis was improved from severe preoperative Grade III (> 80% luminal obstruction) to postoperative Grade II. Breathing was completely normal following two balloon dilations. Patient 6 underwent tracheotomy for severe vocal cord edema on postoperative day 5. The airway at the level of the subglottis was capacious. A postoperative follow-up revealed that the patient had AIDS. During SS-LTR, the patient was in the window period of infection, which led to AIDS being undetected. Vocal cord edema was caused by repeated upper respiratory infection due to AIDS. After the AIDS-related symptoms were alleviated, the patient was successfully decannulated, and that occurred at approximately 1 year following SS-LTR. The current overall decannulation rate was 100% in the present study.

**Table 2:** Postoperative patient characteristics

No.	Preoperative grading	Postoperative grading	Complication	Follow-up, mo
1	III	Normal	None	60
2	III	II	Severe pneumonia	56
3	III	I	None	56
4	II	Normal	None	55
5	III	I	Neck subcutaneous emphysema	54
6	III	Normal	Vocal cord edema	52
7	III	I	None	48
8	III	I	None	47
9	III	Normal	None	44
10	III	I	None	43
11	III	I	None	42
12	III	I	None	37
13	III	I	None	36
14	III	I	Neck subcutaneous emphysema	28
15	II	Normal	None	27
16	III	I	None	27
17	II	Normal	None	23
18	III	I	None	12
19	III	I	None	9
20	III	Normal	None	8
21	III	I	None	8
22	III	Normal	None	3

## Discussion

SGS is one of the most common causes of airway obstruction in children. Significant progress has been made in the treatment strategies for SGS over the past four decades. LTR is the most commonly used open airway reconstruction technique. SS-LTR was developed to reduce the morbidity of postoperative tracheostomy and the long-term placement of an intraluminal stent. Various studies have reported decannulation rates ranging from 82 to 96% following SS-LTR [5, 6, 8]. In the present study, the overall decannulation rate was 100%. In contrast to studies reporting that the postoperative care is administered in the pediatric intensive care unit with an endotracheal tube, the patients in the present study had the endotracheal tube removed immediately following anesthetic resuscitation and were returned to the general ward. Apart from two cases that required reintubation or tracheotomy, all the other cases were discharged successfully. The present study confirmed that extubation immediately following surgery is safe and feasible.

There are a number of advantages to the treatment described herein. First, the treatment used in the present study may reduce the risk of further injury to the surgical site by the endotracheal tube. Previous research has confirmed that 90% of acquired (SGS) cases are caused by prior intubation [9]. Although the incidence of SGS due to intubation has been significantly reduced by several measures, the possibility of airway injury remains as long as the endotracheal tube is present [10–12]. Second, the treatment used in the present study may help avoid the need for sedation during ongoing endotracheal intubation. Sedative and analgesic agents may cause adverse respiratory effects, such as depressed cough reflex and ineffective clearing of secretions, which may lead to infection at the surgical site [13]. Furthermore, abrupt discontinuation of sedative and analgesic agents may result in withdrawal syndrome. 13 In the event of withdrawal symptoms, involuntary vigorous movement of the head may affect the stability of the reconstructed laryngeal structure and cause the potentially life-threatening complication of inadvertent tracheal extubation [14]. Finally, the treatment used herein may avoid the high medical and nursing care costs in the pediatric intensive care, which places a huge financial and emotional burden on families. Therefore, avoiding postoperative endotracheal intubation when possible should be considered a priority in SS-LTR.

For patients with early extubation, reintubation is significantly risky in the early postoperative period (within the first week or 10 days). Our experience is that this can be very difficult and risky. The curve of the endotracheal tube means that the tip of the tube tends to dislodge the graft or impact in the space between the graft and the tracheal incision, leading to failed intubation or the creation of a false passage. Video-assisted laryngoscopy and fiberoptic intubation may be effective methods for the management of the known or anticipated difficult airway [15, 16]. Compared with fiberoptic intubation, video-assisted laryngoscopy intubation takes less time and is suitable for rescue intubation [17]. Fiberoptic intubation is less invasive compared with video-assisted laryngoscopy [18]. The anesthesiologist should have a preformulated strategy for intubation of the difficult airway. This strategy will depend on the condition of the patient, and the skills and preferences of the anesthesiologist [19].

In our series, to avoid damaging the anterior costal cartilage graft, patient 2 was successfully reintubated by fiberoptic intubation on postoperative day 7. But scar tissue hyperplasia was founded at the subglottis. Patient 6 presented with dyspnea on postoperative day 2 due to severe vocal edema. There was no improvement after active treatment. It was considered to be chronic severe vocal cord edema. Inflammation of the vocal cords was unlikely to decrease in a short period of time. A tracheotomy was performed without intubation on postoperative day 5. This indicates that SS-LTR of SGS is not a valuable procedure in patients with significant comorbidities. The presence of significant comorbidities calls for a double-stage surgery [20].

Various conditions must be met before considering SS-LTR without postoperative endotracheal intubation, and these are the following:

i) The site of stenosis is confined only to the subglottis, which does not involve the glottic structures or trachea. The most common glottic lesions include posterior glottic stenosis and bilateral vocal fold immobility (paralysis or cricoarytenoid joint fixation). Younis et al. reported that the overall decannulation rate in the SGS with glottic lesions was significantly reduced compared with the isolated SGS following SS-LTR [21]. When SGS extends up to the glottis, an expansion LTR with posterior cartilage graft has been recommended in the pediatric population [22, 23]. To prevent vocal cord adhesion or scar contracture, the stent should be placed in the laryngeal cavity following reconstruction. The placement of the stent will result in the inability to extubate immediately after surgery. When SGS extends into the upper trachea, partial cricotracheal resection has now been widely accepted as a superior alternative to LTR [24, 25]. Previous studies have reported that the severity of the stenosis determines the surgical staging of LTR [10, 26]. SS-LTR is primarily used for children with less severe SGS (Grade I or II). Pediatric otolaryngologists should not hesitate to resort to a DS-LTR with posterior and/or anterior cartilage grafts supported by an endoluminal stent in the case of severe stenosis (Grade III–IV). However, in the present study, there were 14 cases of severe Grade III SGS (> 80% luminal obstruction). The patients were still successfully extubated after SS-LTR. This suggests that the severity of airway stenosis requires expeditious surgical management to secure the airway and is not the main factor influencing the selection of surgical stage. The site of stenosis may determine surgical staging and the mode of transplantation.

ii) Adequate airway reconstruction must be possible with an anterior cartilage graft to ensure optimal stability of the laryngeal framework. The cricoid cartilage is a complete annular structure. Anterior midline division of the cricoid cartilage alone should not affect the stability of the larynx. If posterior grafting is necessary, then anterior and posterior midline divisions of the cricoid cartilage are required. While the laryngeal field is poorly exposed, the incision must be extended into a full laryngofissure to provide optimal exposure of the cricoid plate for the posterior cricoid split and adequate room for fixing the posterior costal cartilage graft into position. The aforementioned surgery will severely affect the stability of the laryngeal structure. Gustafson et al. reported that the postoperative reintubation rate was twofold and the postoperative tracheostomy rate was threefold higher in the patients who had undergone SS-LTR with anterior and posterior costal cartilage graft compared with single costal cartilage graft [6]. The most important reason for these postoperative outcomes is that the stability of the cricoid cartilage was severely compromised by the anterior and posterior costal cartilage transplantation [6]. A stent of proper diameter and length must be selected after surgery to ensure the stability of the airway structure and prevent the translocation of the graft [27]. The stent will result in the inability to extubate immediately following surgery. Only anterior costal cartilage transplantation was performed without affecting the stability of the larynx. It is recommended that no stent should be placed after surgery. The endotracheal tube can be removed immediately following anesthetic resuscitation and the patient can be returned to the general ward.

iii) The costal cartilage must be used as the anterior graft material for LTR. Many different cartilages (costal, thyroid, auricular and septal) have been used as graft materials [28–31]. The primary advantages of the costal graft are that it can be carved to create flanges. The flanges of the costal graft play a key role in preventing the graft from prolapsing into the airway [27]. However, flanges cannot be created in the other cartilaginous grafts due to their thin profile, which eliminates the ability to buttress the graft against the outer airway for support. Therefore, the endotracheal tube must be placed as a support to prevent the graft from moving into the airway, which also results in the inability to immediately extubate following anesthetic resuscitation. Furthermore, the costal graft is larger compared with the other cartilaginous grafts. The costal graft can be carved to be the widest to provide the most expansion at the level of the stenosis [27]. Adequate expansion is the key to successful surgery.

A limitation of the present study was the lack of cases with Grade IV SGS. Grade IV SGS often involves extended stenosis, and often expanding to the glottis or trachea. For Grade IV SGS, DS-LTR or partial cricotracheal resection are considered as the optimal treatment options [32]. As a result, patients with Grade IV SGS cannot be removed from the intensive care unit immediately following anesthetic resuscitation and returned to the general ward.

In summary, the present study demonstrated that SS-LTR with an anterior costal cartilage graft is an effective method for the treatment of simple SGS ranging from Grade I to III in pediatric patients. Extubation immediately following surgery is considered to be safe and feasible.

## Data Availability

The datasets used and/or analysed during the current study are available from the corresponding author on request.
